# Branded prescription drug spending: a framework to evaluate policy options

**DOI:** 10.1186/s40545-017-0115-9

**Published:** 2017-10-02

**Authors:** Jeromie Ballreich, G. Caleb Alexander, Mariana Socal, Taruja Karmarkar, Gerard Anderson

**Affiliations:** 1 Department of Health Policy & Management, Johns Hopkins Bloomberg School of Public Health, 624 N. Broadway, Baltimore, MD 21205 USA; 2 Center for Drug Safety and Effectiveness, Johns Hopkins Bloomberg School of Public Health, Baltimore, MD USA; 3 Department of Epidemiology, Johns Hopkins Bloomberg School of Public Health, Baltimore, MD USA; 4 Division of General Internal Medicine, Johns Hopkins Medicine, Baltimore, MD USA; 5 Department of International Health, Johns Hopkins Bloomberg School of Public Health, Baltimore, MD USA

**Keywords:** Drug pricing, Pharmaceutical pricing, Drug policy, Pharmaceutical policy

## Abstract

**Background:**

High drug spending is a concern for policy makers due to limits on access for patients. Numerous policies have been proposed to address high drug spending. The existence of multifarious proposals makes it difficult for policy makers to consider all the alternatives. We developed an approach to select the most viable options to present to policy makers.

**Methods:**

We identified 41 different proposals in the peer-reviewed literature to reduce the level of spending or change the incentives for branded prescription drugs; ten of which we identified as promising proposals. Based on criterion used to assess various legislative proposals regarding branded pharmaceuticals we developed a framework to evaluate the ten promising proposals. We then used a modified Delphi technique to iteratively evaluate these ten proposals starting with the initial criterion. During each iteration, five researchers independently evaluated the ten policies based on available criterion and assessed how to modify the criterion to achieve consensus on what attributes the criterion were intended to measure. We highlight areas of disagreement to show where modifications to existing criterion are needed.

**Results:**

We found general agreement for most policy-criterion combinations after three iterations. Areas with the greatest remaining disagreement include possible unintended consequences, the concept of value implied by many of the policies, and secondary effects by the pharmaceutical industry, insurers, and the FDA.

**Conclusions:**

Our analysis provides an approach that can be applied to evaluate policy proposals. It also suggests factors that policy analysts and researchers should consider when they propose policy options and where additional research is needed to assess policy impacts. Developing an objective approach to compare alternatives may facilitate the adoption of policies for branded prescription drugs in the U.S. by allowing policy makers to focus on the most viable options.

**Electronic supplementary material:**

The online version of this article (10.1186/s40545-017-0115-9) contains supplementary material, which is available to authorized users.

## Background

High levels of spending for branded prescription drugs have once again captured the concern of U.S. patients, clinicians, payers and policy makers. According to a recent U.S. government study, spending on prescription drugs increased 12.2% in 2014 [1], straining public sector budgets and causing some private insurers to put an increasing percentage of branded drugs on high cost sharing tiers. Much of the growth in spending is driven primarily by high priced branded drugs [2]. There is growing concern that the high prices are restricting access to branded drugs that have the potential to save lives and reduce morbidity. Perhaps the best example of these newly introduced, highly effective, yet very expensive products are the Hepatitis C drugs [3]. A recent CDC study found that hepatitis C was the infectious disease with the highest mortality rate in spite of the availability of a drug that is nearly a complete cure for most patients [4].

In response to these concerns, policy analysts and researchers have developed a wide range of policy options to address drug spending. Policy options have emerged from academic literature (e.g. see Conti and Rosenthal [5]), trade and professional associations (e.g. see The American Medical Associations drug pricing policy initiatives [6]), and proposed legislation (e.g. see Medicare Prescription Drug Price Negotiation Act of 2015 [7]). The suggested policies impact all aspects of the U.S. pharmaceutical market ranging from altering demand of pharmaceuticals via value-based insurance design to increasing supply with changes to the generic regulatory process.

Given the multitude of policy options available, policy makers should be able to choose and institute appropriate policy; however, there has been no major legislation targeting drug spending in the U.S since the Drug Price Competition and Patent Term Restoration Act of 1984 [8] (informally known as Hatch-Waxman of 1984). One reason may be the discordance of priorities across stakeholders which makes consensus building difficult [9]. Another potential reason behind the lack of policy is that there are too many different proposals for policy makers to compare and build consensus around. A meta-analytic review on individual choices has shown that when individuals are presented with multifarious choices, they find it difficult to choose or sometimes fail to even make a choice [10]. The lack of choice or consensus on a policy can be attributed to the difficulty in comparing the relative merits of any given proposed policy without some existing criterion. The challenge is to develop and then apply criterion that can identify the most promising alternatives for the policy maker to consider. This paper presents promising policies based on a literature review, a framework and criterion to evaluate these policies, and demonstrates how this framework can help policy makers build consensus around promising policies.

## Methods

### Policies under consideration

We conducted a structured literature review with expert opinion to identify 41 policy proposals appearing in the peer-reviewed literature that are designed to reduce branded drug spending in the U.S. (Additional file 1). The technical appendix contains the specific search terms, algorithm, and flow chart describing the search process. Articles identified by this process were reviewed by three researchers (MS, JB, TK) and screened based on their relevance as a policy to reduce branded drug spending. Relevant articles were independently reviewed by two readers and a list of policy options was developed. Reference lists of articles identified by the literature review were also examined for other pertinent articles. We identified 41 policies addressing prescription drug spending in the peer reviewed literature and clustered these policies into five groups: revising the patent (and exclusivity) system; encouraging research to increase development of new drugs; altering pharmaceutical regulation; decreasing market demand; and developing innovative pricing strategies. The five categories were based loosely on the economic fundamentals of the U.S. pharmaceutical market. The category “revision of the patent system” relates to the market protections in place to incentivize an industry with high upfront costs and low production costs. The category “encouraging research” relates to the supply of drugs on the market. Altering pharmaceutical regulation addresses the attributes of the market that contribute to imperfect competition such as regulatory barriers to entry. Decreasing market demand categorizes policies directly affecting demand for drugs. Lastly, the “innovative pricing strategies” category includes strategies that suggests alternatives to the current pricing strategy in the U.S. Simply clustering the various proposals into the five groups could make it easier for policy makers to compare policy options. However, some policy makers may not have preconceived ideas on the best approach and may want to compare all 41 ideas or some subset of them.

Since it was not possible to evaluate all 41 proposals in a single manuscript, we used a consensus process to narrow the list to the ten proposals listed in Table 1. We wanted representation from all five groups since they demonstrate very different approaches and chose at least one option from each group. Within each group, we chose proposals based on our assessment of its ongoing presence in policy discourse. It is important to note that the exact proposals we selected are not crucial to assess the value of our analytic approach. A policy analyst could use this framework, including its criteria, to assess any or all of the remaining 31 proposals or any other proposals in the grey literature.

**Table 1:** Ten policy options

Policy	Description	Assumptions
Use of value-based pricing as a means for setting a fair price of new drugs [28]	In this mechanism, the price of new drugs is set after their effectiveness is compared to other existing treatments for the same indication. Drugs that add significant therapeutic benefits (added value) would be entitled to a premium, where drugs that do not add significant value may be priced at the same levels as the similarly effective existing drugs	Assumes that the premiums for drugs with added value are not as high as to offset the savings from drugs with no added value, but no assumption was made on the level of value accepted in the US.
Strengthening criteria for issuing and protecting patents [29]	Patents are crucial for the drug industry to recoup the R&D investment by providing a drug both market and pricing power. However, the patent system can be manipulated with drug patents “evergreened” with minor reformulations or subtle changes to the technology. A policy that strengthens the criteria for issuing and protecting patents should reduce patent system manipulation by making it harder for company to receive a patent for a minor drug reformulation, thereby preventing a company perpetuating market protection and high drug prices.	Assumes that it would make it harder for drugs that do not add significant value (for example me-too drugs or “evergreening” drugs) to enter the market altogether, or that the patents (or market exclusivity) for the non-beneficial drugs would be shorter.
Shift towards earlier approval, separate regulatory bodies (lethal vs. non-lethal diseases), simplified administrative and application details [30]	The current regulatory system is designed to require drugs achieve high standards of safety and efficacy; however, these high standards come with a high cost associated with regulatory burden. This policy would take into consideration a drug’s target health condition and early trial attributes when developing a roadmap to regulatory approval. For example, drugs that target a high severity condition with limited treatment options could be granted approval or conditional approval with fewer required clinical trials. By reducing the regulatory burden for some drugs, this policy would lower the cost of drug development.	Assumes the gains with expediting processes related to severe, life threatening treatments would be higher than the losses from delaying the approval processes for other non-severe, non-life threatening therapies.
Increasing regulatory thresholds so as to increase value of products upon market entry [31]	The current regulatory system does not consider a drug’s comparative effectiveness in the approval process. A policy requiring consideration of a drug’s comparative effectiveness in the approval process increases the regulatory thresholds for drug to achieve approval, but it also limit market entry of drugs that offer no improvements in clinical effects to current drugs on the market. Currently, some drugs enter the market at high prices while offering no improvement in effectiveness. [32] Ineffective drugs do nothing to better patients care and only drive up drug costs.	Assumes that it would make it harder for drugs that do not add significant value (for example me-too drugs or evergreening drugs) to enter the market altogether, or that the patents (or market exclusivity) for the non-beneficial drugs would be shorter.
Adopting episode-based payments for physician administered drugs in Medicare [15]	Medicare currently reimburses physician-administered drugs using the ASP plus 6%. This reimbursement structure incentivizes physicians to administer drugs with the highest ASP since there profits are linear to the drug’s selling price. Researchers have advocated changing the reimbursement to episode-based payments, which incentive physicians to maximizing the clinical care within a set budget.	Assumes that physicians will respond to the modified incentives by reducing the utilization of highly- expensive drugs and favoring other lower cost alternatives. Assumes there will be lower cost alternatives that can be used in order to control the expenditures.
Adopting value based insurance designs that alter coverage based on price, effectiveness, safety and other parameters [33–36]	Recognizing the potential that a formulary has on influencing prescribing and utilization behavior, a policy many researchers have the forward is to encourage wider adoption of value-based insurance designs. Fundamentally, a value-based insurance design uses formulary structure including co-pay, coinsurance, and deductibles to steer patients into choosing drugs that offer to patients the most value.	Assumes that patients and providers will respond to the incentives by increasing the use of value drugs and decreasing the use of non-value drugs. It is possible that manufacturers may reduce prices in order to increase the value of their drugs.
Implementing risk-sharing contracts to ensure upside to pharmaceutical innovators and to protect payers against payments that do not return value to patients [37, 38]	A justification for high drug prices is often a drug’s clinical benefit; however, there has to be skepticism on a new drug’s claimed clinical benefit since much of the clinical benefit data is sourced from phase 3 trials which have strict inclusion/exclusion criteria and do not consider a drug’s tolerability or ease of use. To adjust for the risk of a drug’s claimed clinical benefit, a proposed policy for is the use of a risk-sharing contract between a drug manufacturer and a payer that sets specified clinical outcome targets as required for payment. This protect payers against for paying for drugs that do not offer clinical value or whose clinical value is less than promised.	Assumes risk-sharing contracts will promote utilization of drugs with hypothesized large clinical benefit in lieu of both lower cost drugs and the counterfactual that drugs with large clinical benefits would be automatically covered.
Empowering federal government to negotiate prices for Medicare, Medicaid, VA, PHS, DOD at one price [39–41]	Researchers have attributed high drug prices in part due to the inability of some major payors to negotiate drug prices. Researchers have suggested changes, including legislative changes, to allow the government purchasers of drugs, representing millions of patients, the ability to collectively negotiate prices using, for example, collective negotiation across the five big government purchasers and pharmacy benefit management tactics such as formulary exclusivity.	Assumes manufacturers will respond to the price negotiations with lowering the prices of their products. It is possible that prices will have different levels of decline across the multiple government programs.
Allow drug coupons only for branded drugs with no generic competitor or require disclosure of costs of drugs or alternative treatments [42]	While pharmaceutical companies offering drug coupons to patients appears benign on the face, drug coupons break-down the existing economic incentives that steer patients to potentially lower-priced or higher value drugs. Drug coupons are provided to patients, essentially reimbursing the patient for any cost-sharing. However, cost-sharing is one of the few tools that insurers used to steer patients to lower-priced or higher value drugs.	Assumes that physicians and patients will be sensitive to the price information and will choose to reduce utilization of a more expensive drug if there is a cheaper, similarly effective alternative. Assumes that removing drug coupons from drugs with generic alternative will spur competition and reduce prices.
Incorporating price information into clinical workflow to increase clinician and patient cost sensitivity [29, 43]	Across all of healthcare, there are calls for increased price transparency. The US healthcare market is notoriously fragmented and the fragmentation allows for market distortions such as wide price variation for products or services even though the price of inputs is the same. Incorporating price information into clinical work flow is a way of improving price transparency, an improved price transparency has been suggested to lead to better healthcare decisions.	Assumes that patients and providers will be sensitive to the price information disclosed. Assumes that the disclosed price information will be reflective of the true price for that patient, will be updated with sufficient frequency and will be available across the multiple insurers and government programs.

### Criteria to evaluate policies

The criteria we initially developed were based on our reading of the peer-reviewed literature; the arguments that individuals have made to support their proposals in the literature; discussion in the lay press, trade, and Congressional testimony. For example, a peer-reviewed article discussed framework on assessing policies targeting research and patents [11] while another highlighted challenges for policies to reduce spending [12]. These articles provided suggestions as to areas where criteria should be sensitive enough to assess the pros and cons of proposed policies. From recent congressional hearings, a Chief Executive Officer of a branded pharmaceutical company emphasized the importance of Research & Development (R&D), the Administrator of the U.S. Food and Drug Administration (FDA) stressed the role of regulation, and an executive of a Pharmacy Benefit Manager (PBM) company discussed the market dynamics of the US drug market [13]. We incorporated the concerns of each of these stakeholders into our initial criterion.

However, the criterion that were proposed were worded differently and often had slightly different meanings. We used a Delphi technique to attempt to reconcile the differences and to define criterion with unambiguous meanings. We also set the status quo as a reference point such that our evaluation using the criterion would suggest how a policy changes the U.S. drug market-this approach results in our criterion being value-laden. The criterion evolved as we evaluated each of the ten options and attempted to reconcile our different assessments.

We first present the final nine criterion and then discuss how they were developed. Refer to Table 2 for criterion.

**Table 2:** Nine proposed criteria to evaluate policy proposals addressing the high price of branded prescription drugs in the U.S

Criterion	Rationale
Incentivize drug companies to invest in research & development	We assume that policies that increase manufacturer revenues will increase levels of R&D.
Promote R&D and marketing of high value drugs	Certain drugs provide greater value than other drugs as measured by things incremental cost effectiveness ratios or quality adjusted life years.
Encourage uptake of high value products	Policies that encourage uptake of high value drugs by promoting value decision making by patients, physicians, and payer.
Reduce financial barriers to drugs	Policies that reduce drug prices can facilitate greater access to drugs by reducing the financial burden of drugs.
Lower the overall spending on drugs and medical care	Policies that lower drug prices; reduce drug utilization; or give providers the proper incentives to substitute drugs for other clinical services will lower spending overall and for drugs
Administrative burden	We assume that policies requiring the FDA or companies to perform additional tasks will add to the administrative burden.
Facilitate entry to generic market	Policies encouraging providers to choose the less expensive generic can increase demand of generics on the market
Requirement for legislation	We assume any additional legislative change will prove difficult and policies significantly impacting the pharmaceutical industry will be most difficult to enact.
Potential for unintended consequences	We assume that there will always some level of unanticipated economic or clinical consequences that result from policies that change the rules or alter stakeholder incentives.

Our first criterion assesses the *potential effect of a policy on investment in pharmaceutical research and development (R&D)*. Policymakers recognize the inherent trade-off between technological progress and drug prices [14]. This criterion is designed to reflect the strong support for pharmaceutical innovation.

The second criterion assesses *whether the policy encourages the development of innovative drugs*. Unlike the first criterion, which evaluates policies’ influence on the number of drugs in development, this criterion assesses the level of innovation in drug development. Given the concerns that some new drugs do not offer substantial clinical benefits over existing therapies [15], this criterion evaluates the potential for a policy to encourage the development of more innovative products.

The third criterion evaluates *how well a policy promotes uptake of high value drugs*. During our iterative assessment process, we learned that incentivizing development of new drugs and incentivizing appropriate use of drugs are different aspects of the drug-to-market process. We crafted this criterion to align with policymakers concerns around the uptake of high-value healthcare including drugs by providers, patients and payers [16]. We define “high value” in a cost-effectiveness context as a drug offering a compelling incremental cost-effectiveness ratio.

The fourth criterion evaluates *the potential impact a policy has on patients’ financial barriers to accessing drugs*. Access has proven to be challenging for many patients especially for specialty drugs. This criterion assesses the ability of proposed policies to ensure a patients’ access to needed medications [17].

The fifth criterion evaluates *a policy’s effects on lowering overall drug and medical care spending*. Some prescription drugs have been demonstrated to be substitutes for other medical care thereby offsetting other, potentially avoidable, medical costs [18]. Policy makers may want to recognize the possibility of a trade-off between high drug spending and lower medical care spending.

The sixth criterion targets *a policy’s role in facilitating generic drug utilization*. Branded (on-patent) drugs are typically priced much higher than generic (off-patent) drugs. Policymakers are interested in drug policies encouraging the use of generics [19], thereby, lowering spending-and this criterion assesses policies in this domain.

The seventh criterion evaluates *administrative burden for manufacturers and the FDA*. There is widespread concern that the queues in the FDA evaluation process are restricting timely access to drugs and that drug manufacturers have to spend significant funds on R&D [20]. Mechanisms that facilitate the regulatory process are of great concern to policy makers as long as they do not compromise safety and efficacy.

The eighth criterion examines *whether a proposed policy will require legislation*; legislation is a potentially major hurdle for policy enactment. Policies that require regulatory or administrative changes may be more feasible to enact than policies requiring legislation.

The ninth and last criterion assesses the *risk of unintended consequences*. Incremental policies or policies with larger bodies of supporting evidence tend to have lower risk, as opposed to larger untested reforms that have been shown to unravel once implemented [21]. Policies that have not been implemented in other settings; whose research is limited; or those that radically change current policies and practices pose a higher risk of unintended consequences.

It is possible to weight the criterion, but we did not elect to do this. We expect that some policy makers will place greater weight on controlling drug spending while others may place a higher emphasis on innovation or access. Because we did not assign weights, we computed overall scores for the ten alternatives, and overall scores reflect predicted policy effect and is not representative of strength of evidence nor magnitude of effect.

### Policy assessment and evaluation

We used a modified Delphi technique to analyze the ten proposals [22]. Our team represented different areas of academic expertise including clinical medicine, health economics, pharmacoepidemiology, and health policy. However, all of the participants are academicians at a single academic institution. Our approach could be replicated with a larger and more diverse group of stakeholders and perhaps other criterion would evolve.

Our approach required three iterations in order to reach near consensus. During each iteration, all five members of the team independently evaluated the ten policies. Following the evaluation, results were compared and discussed. Discussions focused on areas of disagreement, with the purpose of understanding the reasons for the differences and adjudicating differences. Some disagreements were the result of differing interpretations of criterion, in which case revisions of criterion were undertaken. Revisions were constructed to reduce the uncertainty around the scope of a criterion before proceeding with the next round of assessments.

For the first seven criteria, policies were scored either “worse than status quo”, “status quo”, or “better than status quo”. The eighth criteria, examining the need for legislation, was evaluated as either “requiring legislation” or “not requiring legislation”, while the last criterion, the potential for unintended consequences, was scored as or “low”, “medium” or “high”.

## Results

### Preliminary review of each policy

**Fig. 1:**
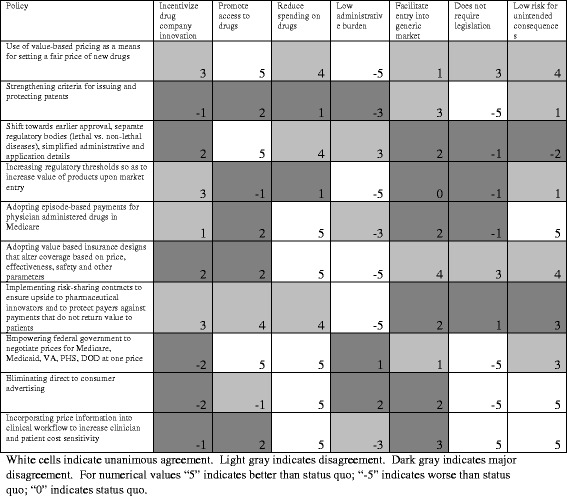
Results of first round evaluating policies to reduce spending of branded prescription drugs in the United States

We began with seven criterion gleaned from a variety of sources. Figure 1 depicts the results of the first round of analysis that we conducted. White boxes reflect areas where high levels of agreement were present; grey boxes represent areas where there was moderate agreement; and black boxes represent areas where there was low agreement. The numerical scores within each cell reflect the cumulative scores across the 5 reviewers, with a score range of −1 to 1 for each reviewer. For example, the use of value-based pricing received a score of 3 for the criterion “*incentivize drug company innovation”*. Although not seen on the matrix, four reviewers scored the policy of using value-based pricing as “better than status quo” for this criterion, and one reviewer scored the policy “worse than status quo”, with the final score being +4 for “better than status quo” and −1 for “worse than status quo” for a total score of 3. Through the Delphi process we attempted to understand the reasons for the disagreement and revised the criterion.

As depicted by Fig. 1, in the first round approximately one-fourth (27.1%) of the seventy policy-criterion combinations had total agreement, while the remainder reflected either general agreement (32.9%) or disagreement (40%). We focused on the reasons for disagreement.

Much of the disagreement during Round 1 was about the meaning and wording of certain criterion. This suggests areas where the criterion is most uncertain and could foster the most policy debate. For example, an initial criterion was whether a specific policy *incentivizes drug company innovation.* Some interpreted this criterion as whether a policy increases R&D, while others interpreted this criterion as whether the policy shifts focus to developing truly innovative drugs rather than “me-too” drugs or “low-value” drugs. As a result, this one criterion was broken into two in order to achieve greater consensus. Similarly, the criterion *Reduce drug spending* raised concerns about whether this criterion was too general and missed the reduction of offsetting medical care, an argument raised in the justification of high priced hepatitis C drugs [23]. As a result of this first analysis, we clarified the definitions of each criteria and expanded the number of criteria from seven to nine.

### Intermediate review of each policy

**Fig. 2:**
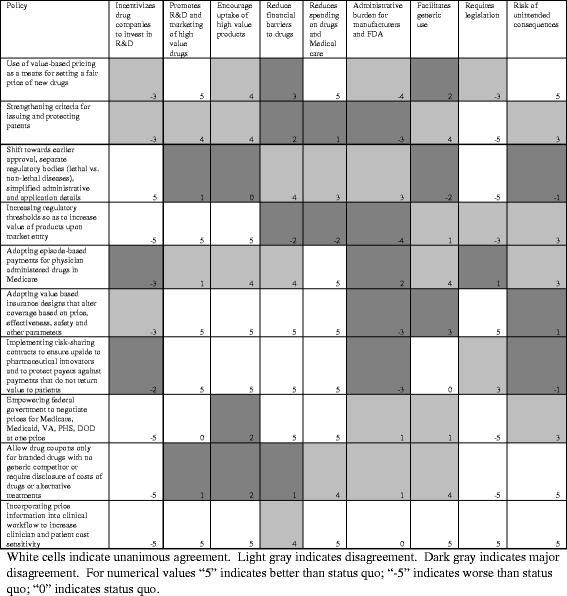
Results of second round evaluating policies to reduce spending of branded prescription drugs in the United States

Figure 2 depicts the results of our second round of analysis. In this round, approximately two-fifths (40%) of the policy-criterion combinations had total agreement, while 32.2% reflected general agreement and 27.8% reflected disagreement. While disagreements in the first round focused primarily on the interpretation of the criterion, disagreements during the second round generally focused on the projected effects of each policy (e.g., “to what degree will changes in FDA regulatory processes towards earlier approvals encourage the uptake of high value products”?). Revising criteria addressed much of the first round disagreement, the nature of the second round disagreements could not be fully addressed with criterion revisions; rather we attempted to address disagreements by discussing anticipated policy effects.

### Final review of each policy

**Fig. 3:**
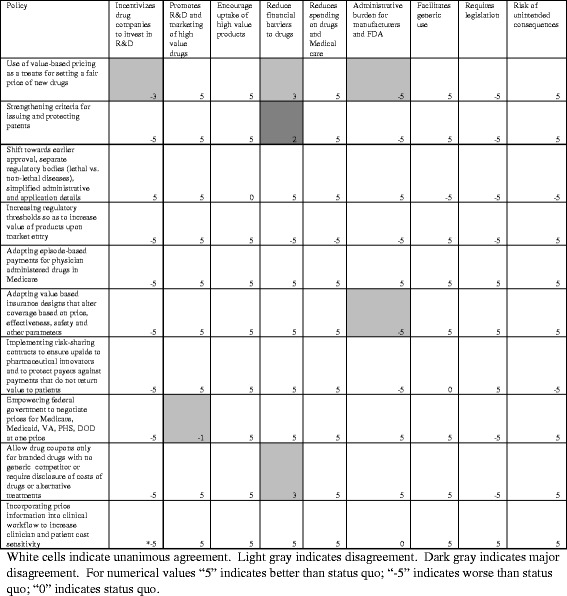
Results of final round evaluating policies to reduce spending of branded prescription drugs in the United States

Figure 3 depicts the results of our final analysis. Through discussion of each researcher’s position and the current literature, we reconciled most disagreements observed during the second round. However, there was still disagreement in 7.8% of the policy-criterion combinations, driven primarily on disagreements over unintended consequences, the concept of value implied by many of the policies, and secondary effects by the pharmaceutical industry, insurers, and the FDA. Based on overall, unweighted scores, the three highest scoring policies are: adopting episode based payments, adopting value-based insurance design, and incorporating price information in the workflow.

## Discussion

Many proposals have been proposed to reduce branded drug spending in the U.S., and we focused on those in the peer-reviewed literature. Multifarious proposals make it difficult for policy makers and other stakeholders to take action. To help policy makers build consensus around promising policies, we developed an evaluation framework that allows policy makers to make such comparisons, and used a modified Delphi technique to apply this framework to a select sample of ten policies.

From the literature, we found little direct research on how policy makers should build consensus around promising policies to address branded drug spending in the U.S. Some international studies have examined the priorities of key stakeholders and policy makers, and suggest consensus building based on alignment of values [9, 24]. Others have discussed specific policies and their pros and cons of these policies [25]. While both approaches have merits in identifying stakeholder preferences and individual policy trade-offs, neither approach lends itself for an objective evaluation across multiple policies.

Our analysis demonstrates that it is possible to use a Delphi technique to revise the criterion used to assess alternative policies to minimize areas of disagreement. A limitation of this approach is generalizability; it is clearly possible that a different group would develop different criterion. However, our initial list is based on the arguments made by various individuals to support their proposals; discussion in the media, and trade and Congressional testimony. Early disagreement during Round 1 and to a lesser degree, Round 2, regarding the explicit criterion to use and the wording gave way to disagreements in the later rounds regarding the long run implications of specific policies and secondary effects of policies. A major benefit of this approach is that it can identify where there is disagreement on the criterion used to evaluate various options and help researchers developing policy alternatives to consider each criterion when the develop policy alternatives. Many important issues are not discussed in many policy proposals.

One important area of disagreement was related to the concept of “value” both regarding value-based pricing policies and value-based insurance design. When the authors developed policies promoting value-based practice, it was assumed that a value-premium or value-threshold would exist, thereby demarcating high-value versus low-value. However, without knowing specific details of a policy implementation and how value is defined, i.e. from whose perspective, what level, etc., the concept of “value” was a source of considerable disagreement among the five academics. Researchers and policy analysts developing alternatives may want provide better definitions of value.

The ambiguity around “value” hindered consensus around the effect of policies such as value-based purchasing. One argument was whether value premiums would be set at levels similar to those observed in Europe and the lower drug prices paid in Europe are in part due to the different level of value premiums [26]. However, others argued that a combination of the strength of the pharmaceutical lobby and fragmented payor market would result in US value premiums being set higher than Europe, and drug prices would be set to the maximum threshold. There was also disagreement concerning whether certain policies would increase administrative burden for manufacturers and FDA. Consider, for example, how value-based insurance design would impact manufacturers and the FDA: would manufacturers be more inclined to conduct phase 4 or pragmatic clinical trials testing the effectiveness rather than efficacy in order to demonstrate value? These issues require more discussion in the policy proposals.

There was residual disagreement regarding the secondary effects of proposed policies, especially the response from the pharmaceutical industry. For example, authors disagreed over whether strengthening criteria for issuing patents would reduce the likelihood of “evergreening” of existing patented drugs by the drug companies [27], thereby, facilitating a more robust and lower cost generic market. Alternatively, strengthening criteria could limit “me-too” drugs that act as competitors, and the reduction of competition would ultimately drive up prices.

Further disagreement centered on the potential secondary effects of some of the policies on payers, and in turn, patients. For example, consider a policy such as value-based pricing. While there was general agreement that this type of pricing would encourage the development of high-value, innovative drugs, there were differing opinions as to whether changes to cost-sharing structures and drug prices would result in price increases for patients. A net increase in price exposure would increase the financial barrier to drugs.

The last theme of disagreement was the uncertainty regarding potential unintended consequences. The problem is that for some of these policies it is difficult to develop a control group since it is an all or nothing policy change. For example, we could only speculate on the nature of unintended consequences, due to the difficult assessing policies in largely hypothetical scenarios. For example, would an unintended consequence of requiring comparative effectiveness information on newly approved drugs significantly increase development costs? Alternatively, what value premiums are acceptable in the US and how would adoption of specific value premiums affect aggregate drug spending, and the impact of value premiums on patient, prescriber, and payer behavior.

Considering the motivation of the study was to provide a framework to assess policies aimed at reducing branded drug spending and not to suggest specific policy solutions, our limitations focused on the methodology. There were three main areas of limitations: selection of criteria, evaluation of policies, and generalizability. First, we attempted to identify the best criterion for a national policy discussion, however, our criterion may reflect our perceptions as five members of an academic institution. Other policy analysts may have different priorities and by extension criterion. The second main limitation is the evaluation of each criterion-policy pair. For most policy proposals, researchers tended to keep the proposal vague, which makes it difficult to assess the effects. Case in point is the policy to allow the federal government to negotiate drug prices. The U.S. Congressional Budget Office suggests price negotiations a range of effects depending on the circumstances of the negotiation [24]. Knowing the details of a negotiation policy could improve the confidence of the effects of this policy. Another limitation related to policy evaluation is the single policy approach for the evaluation. It is possible policies are not substitutes but rather complements, thus the effect is dependent on which policies to include. It is also realistic to assume any national policy will include multiple policies and there could be trade-offs across policies. The last limitation is generalizability, which cuts across multiple aspects. As suggested earlier, our criterion may not be generalizable to non-academic institute policy analysts. Evidence for policies chosen may not be generalizable to the U.S. national policy. Lastly, our framework may not be applicable to other countries.

## Conclusions

Policy makers have a difficult time when there are multifarious policy options that address different aspect of the policy debate with different approaches. This makes it less likely for them to take action. An approach that allows policy analysts to use explicit criterion to examine the alternatives to reduce the number of available option is needed. Whenever possible, a comparison based on explicit and objective criterion is needed. In order to accomplish this there needs to be agreement on how to assess the criterion.

In this analysis of policy options to reduce spending of branded prescription drugs in the U.S., we found general agreement for most policy-criterion combinations. It required three iterations to reach general agreement starting from criterion initially gleaned from Congressional testimony, our reading of the peer-reviewed literature; the arguments that individuals have made to support their proposals in the literature; discussion in the lay press, and trade press. However, after three iterations disagreement persisted and the areas of continued disagreement suggest topics of further research especially empirical data to improve our understanding and quantification of the potential real-world effects of these policies.

Our findings also underscore the importance of clear definitions of the policies and their components as well as the importance of considering the primary and secondary outcomes of policies. Researchers developing policies need to consider the criterion that policy makers may use to assess the alternatives such as a policy’s effect on research and development and whether it drives innovative medicine development or “me-too” drug development. Even with consensus on the direction of effect for a policy, our analysis suggests that the overall net effect of policies may be difficult to estimate, in part because of differences of opinion in their short versus long-term implications. This suggests areas for additional research. While uncertainties could increase the likelihood that policy makers will choose the status quo, pressure for change is building and policy makers will be reviewing the available options. More analysis on the unintended consequences of various alternatives is necessary. For a problem with a multitude of potential policy solutions, an approach such as we propose that focuses on the criterion that will be used to evaluate the options may be helpful. At a minimum, it suggests criterion that policies should address and points out areas where there is the greatest uncertainty regarding policy change.

## Additional file

Additional file 1: Policy identification process. (DOCX 31 kb)

## Abbreviations

CDC: Center for Disease Control
FDA: Food and Drug Administration
PBM: Pharmacy Benefit Manager
R&D: Research and Development
