# Supplementary material for: Branded prescription drug spending: a framework to evaluate policy options
Source: J Pharm Policy Pract. 2017 Oct 2;10:31. doi: 10.1186/s40545-017-0115-9 (PMC5625822; doi:10.1186/s40545-017-0115-9)
Supplement: Additional file 1: — Policy identification process. (DOCX 31 kb) [file 40545_2017_115_MOESM1_ESM.docx]

**SUPPLEMENTAL MATERIAL: POLICY IDENTIFICATION**

We identified a range of peer reviewed policy options that aim to reduce branded drug spending in the U.S. using a two-step process. First, we convened a small group of experts in the field and had them identify policy options that are available to reduce either branded drug costs or spending. Their discussion identified seminal articles. Based on these seminal articles, we constructed a preliminary list of policy options and key words that served as a format for conducting the literature review to identify additional policy options. Second, we conducted a literature review as described below. Articles identified by both processes were reviewed by three researchers (MS, JB, TK) and screened based on their relevance as a policy to reduce branded drug costs. Relevant articles were independently reviewed by two readers and a list of policy options was developed. Reference lists of articles identified by the literature review were also examined for other pertinent articles. We identified 41 policies addressing prescription drug spending in the peer reviewed literature.

**Technical appendix**

**1. Keywords**

**CONCEPT 1: DRUG**

(Drug OR drugs OR pharmaceutical OR pharmaceuticals OR "pharmaceutical products" OR medicine OR medicines)

**CONCEPT 2: PRICING**

(Price OR prices OR pricing)

**CONCEPT 3: REFORM PROPOSAL CATEGORY:**

**3.1 Patent Laws**

("patent* law*" OR " patent* legislation" OR " patent* length") OR ("market exclusivity" OR "market access" OR "market share")

**3.2 Regulatory Process**

("comparative effectiveness" OR reimportation OR "payment system*" OR "regulatory process*" OR competition OR monopoly OR ((closed OR controlled) AND ("distribution system*")) OR "Bayh-dole act")

**3.3 Coverage Policies**

("pric* transparency" OR "preferred pharmacy" OR "drug* formulary" OR "tiers" OR "deductible" OR "out-of-pocket" OR "copayment" OR " copayment offset programs" OR "coupons" OR "cost-sharing" OR "specialty drug*" OR "specialty pharmacy*")

**3.4 Pricing Policies**

("Price* negotiation*" OR "pricing* negotiation*" OR rebate* OR "shared discount*" OR "wholesale margin*" OR "risk sharing" OR "cost-sharing" OR "bundle payment*" OR "fair pric*" OR "share of rebates from manufacturer*" OR chargeback OR "reference pric*" OR "price* gouging")

**3.5** **Changing Demand**

("direct-to-consumer advertising" OR advertising OR marketing OR "consumer information")

**3.6 Research Generation**

(R&D OR "research and development" OR "drug discovery" OR "value-based" OR "clinical trial*")

**CONCEPT 4: INTERVENTIONS**

(Reform OR law OR legislation OR policy OR policies OR strategy OR strategies OR intervention OR interventions)

**CONCEPT 5. ACTORS**

(CMS OR Medicare OR PBM OR "pharmaceutical benefits manager*" OR Medicaid OR "health insurance" OR "insurance compan*" OR "pharmaceutical industry" OR "FDA" OR patient* OR consumer*)

**2. SEARCH STRATEGY**

- Concepts 1 + 2 + 3 + 4 + 5 combined by "AND" statements
- Concept 3= sub-concepts 3.1-3.6 combined by "OR" statements
- Note: the search strategy screened title, abstract, original title, name of substance word, subject heading word, keyword heading word, protocol supplementary concept word, rare disease supplementary concept word, and unique identifiers.

**Flowchart OF ARTICLE SCREENING**

Screening: 3 independent researchers

Pubmed:

343 articles retrieved

Full-text review: 3 independent researchers

Final article selection:

N=22 articles

Selected articles:

N=40 articles

Not selected articles:

303 Non-USA or no policy intervention

Not selected articles:

18 no policy intervention or not sufficient depth in description
